# Epigallocatechin Gallate Ameliorates Nicotine Withdrawal Conditions-Induced Somatic and Affective Behavior Changes in Mice and Its Molecular Mechanism

**DOI:** 10.1155/2023/5581893

**Published:** 2023-06-13

**Authors:** Mahardian Rahmadi, Ahmad D. Nurhan, Retno I. A. Rahmawati, Theresia F. Damayanti, Djoko A. Purwanto, Junaidi Khotib

**Affiliations:** ^1^Department of Pharmacy Practice, Faculty of Pharmacy, Universitas Airlangga, Surabaya 60115, Indonesia; ^2^Biomedical Pharmacy Research Group, Faculty of Pharmacy, Universitas Airlangga, Surabaya 60115, Indonesia; ^3^Department of Pharmaceutical Sciences, Faculty of Pharmacy, Universitas Airlangga, Surabaya 60115, Indonesia; ^4^Pharmaceutical Analysis Research Group, Faculty of Pharmacy, Universitas Airlangga, Surabaya 60115, Indonesia; ^5^Biomaterial Translational Research Group, Faculty of Pharmacy, Universitas Airlangga, Surabaya 60115, Indonesia

## Abstract

In nicotine withdrawal (NW) conditions, molecular changes, such as increasing corticotropin-releasing factor (CRF) in the amygdala, and melanocortin signaling in the hypothalamus, can occur in the brain, leading to increased feeding behavior and body weight as somatic changes as well as high anxiety-like behavior as an affective changes. Therefore, this research aimed to investigate the effect of epigallocatechin gallate (EGCG), the largest component in green tea, on CRF, pro-opiomelanocortin (POMC), and melanocortin four receptor gene expression in the brain under NW conditions. The 24 Balb/c male mice used were randomly divided into four groups. The doses used included normal saline 1.0 mL/kg as a control group, and nicotine 3.35 mg/kg that was administered subcutaneously three times a day. After NW conditions, EGCG 50 mg/kg was administered intraperitoneally two times a day. Behavior evaluation was performed to measure somatic and affective changes, and the animal was sacrificed for molecular analysis. The results showed that NW conditions significantly increased food intake, body weight, and anxiety-like behavior compared with the normal group. Meanwhile, EGCG significantly decreased food intake, body weight, and anxiety-like behavior compared with NW conditions in mice without EGCG. The polymerase chain reaction results also showed that EGCG decreased the CRF mRNA expression in the amygdala and increased the POMC. This indicated that EGCG improved somatic and affective behavior in NW conditions by decreasing CRF mRNA expression in the amygdala and increasing POMC mRNA expression in the hypothalamus.

## 1. Introduction

The devastating effects of tobacco smoking on human health are well-established. It is one of the leading causes of mortality worldwide, with approximately 5 million deaths attributed to it each year [1]. Smoking can have a significant impact on various aspects of health, including increasing the risk of several medical complications. Studies have shown that smoking can increase the risk of diabetes, breast cancer, lung disease, heart complications, and kidney disease [2].

Nicotine, the primary component in cigarettes, is notorious for causing addiction by activating the nicotinic acetylcholine receptor (nAChR) [3]. In the central nervous system, nicotine binds to nAChR in the ventral tegmental area. It stimulates the release of the neurotransmitter dopamine by dopamine neurons in the nucleus accumbens (NAc), which results in the emergence of reward nicotine effects, such as euphoria [4].

Sudden discontinuation of nicotine in chronic smokers causes a symptom known as nicotine withdrawal (NW) conditions. These changes appear at 4–12 hours and peak on the third day after cessation of chronic nicotine use. The symptoms of NW conditions are characterized by somatic, affective, and cognitive changes [5]. Somatic changes are physical, which include tremors, bradycardia, and increased appetite. Affective changes related to emotions, such as anxiety, depression, dysphoria, hyperalgesia, and irritability. Furthermore, cognitive changes include difficulty concentrating and memory impairment after chronic NW conditions [5, 6].

Chronic NW conditions cause dysregulation of brain regions associated with stress and the emotional system. According to a previous report, corticotropin-releasing factor (CRF) plays a central role in developing negative affective states during withdrawal [7]. This is because the activation of the CRF system produces anxiety-like behavior in the absence of medication. CRF is an essential regulator of the stress system that drives addiction-like behavior by activating the hypothalamic–pituitary–adrenal (HPA) axis and the extended amygdala [7, 8]. However, changes in the CRF system can occur in the structures of the extended amygdala, including the central nucleus of the amygdala (CeA), and the NAc [7, 8].

According to a recent research, the expression of pro-opiomelanocortin (POMC) mRNA in the arcuate nucleus (ARC) of the hypothalamus has been increased due to the activation of *α*3*β*4 nAChR by nicotine [9]. POMC is a precursor peptide of the anorexigenic neuropeptide *α*-melanocortin stimulating hormone (*α*-MSH), which binds and activates the melanocortin four receptor (MC4R) in the paraventricular nucleus (PVN) of the hypothalamus. Therefore, under NW conditions, decreased POMC leads to reduced *α*-MSH release and low MC4R function in the PVN. Weight gain is also influenced by the role of agouti-related protein (AgRP) in hypothalamic ARC, which is an endogenous antagonist of MC4R [10]. The phenomenon of weight gain in NW conditions is also influenced by POMC, MC4R, and AgRP, the melanocortin system in the hypothalamus. This melanocortin system is critical in regulating energy balance, food intake, energy expenditure, and body weight in humans [11].

**Figure 1:**
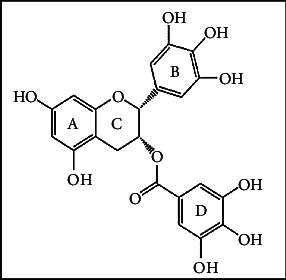
The chemical structure of (−)-EGCG [12].

Epigallocatechin gallate (EGCG; Figure 1) is a catechin polyphenol group widely found in green tea (*Camellia sinensis*) and constitutes 59% of the catechin contents [13]. EGCG exhibits pharmacological effects, such as antioxidant, anti-infection, anti-inflammatory, anti-cancer, cardioprotective, anti-obesity, and other complications of metabolic syndrome [11, 14–20]. Furthermore, it produces neuroprotective and neurodegenerative effects against toxins, depression, memory loss, and stress-induced behavior disorders [21, 22].

Mechanism of EGCG as an anti-infection by L-pneumophila bacteria due to nicotine is through macrophages that produce tumor necrosis factor alpha (TNF-*α*) and interferon gamma (IFN-*γ*) [15]. EGCG also inhibits reactive oxygen species (ROS) activated by nicotine, resulting in anti-cancer and cardioprotective effects by increasing the activity of antioxidant enzymes [18, 23]. Based on this research, EGCG is known to prevent a decrease in BDNF, indicating that the compound inhibits stress-induced HPA axis dysfunction [20]. The compound also decreases food intake by increasing POMC mRNA expression in the hypothalamic ARC [24]. This reveals that EGCG can inhibits affective and somatic changes in NW conditions through mechanism of reducing stress and appetite.

## 2. Materials and Methods

### 2.1. Experimental Animals

The Balb/c male mice aged 2–3 months, with body weight 20–30 g and a healthy physical conditions used in this research were obtained from Surabaya, Indonesia. Mice were placed in cages with a temperature of 24 ± 1°C, with lighting for 12 hours on a light cycle and 12 hours on a dark cycle. All experimental animals were habituated in a rearing environment for seven days before treatment. This research was conducted at the Animal Laboratory Research Center, Faculty of Pharmacy, Universitas Airlangga, Surabaya, Indonesia. All protocols were approved by the research ethics committee of the Faculty of Veterinary Medicine, Universitas Airlangga, Surabaya, Indonesia (Animal Care and Use Committee), with research ethics certificate No. 2.KEH.050.03.2022.

### 2.2. Drugs and Experimental Design

EGCG (Xi'an Rongshen Biotechnology Co., Ltd., China) with a purity of ≥98%, nicotine (Sigma–Aldrich, USA), and NaCl 0.9% (PT. Otsuka Indonesia) were used in this research. Before the treatment, EGCG and nicotine were dissolved in normal saline (NaCl 0.9%) in a recenter apparatus.

**Figure 2:**
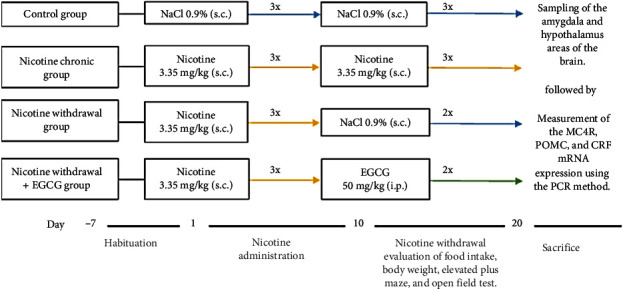
Experimental design scheme.

A total of 24 mice were randomly divided into four groups (*n* = 6) consisting of (1) normal group, where mice were injected with normal saline 1 ml/kg subcutaneously (s.c.) three times daily for 20 days; (2) nicotine group, mice were injected with nicotine 3.35 mg/kg s.c. three times daily for 20 days; (3) NW conditions group, mice were injected with nicotine 3.35 mg/kg s.c. three times daily for 10 days, followed by normal saline 1 mL/kg s.c. two times daily for 10 days; and (4) NW condition + EGCG group, mice were injected with nicotine 3.35 mg/kg s.c. three times daily for 10 days, followed by EGCG 50 mg/kg intraperitoneally (i.p.) two times daily for 10 days. Schematically, the experimental design was shown in Figure 2.

In previous research, the chronic nicotine model was developed in mice by treatment with 3.35 mg/kg nicotine s.c. three times daily for 7 days [25]. However, this research used a chronic nicotine treatment model by administering nicotine 3.35 mg/kg s.c. three times daily to mice for 20 days. There were still no research regarding the effects of EGCG on NW. Therefore, EGCG dose of 50 mg/kg used in this research was based on a recent report, where the 50 mg/kg dose i.p. two times a day in mice effectively reduced the reward effect caused by nicotine [26].

### 2.3. Somatic Response Examination

Somatic response parameters observed were food intake and body weight profiles on NW conditions. After 24 hours of cessation of chronic nicotine administration or during NW, the amount of food consumed by the experimental mice was weighed daily to record the food intake profile. The experimental animals were weighed each day in the morning before treatment administration.

### 2.4. Affective Response Examination

Affective response parameter observed was the presence of behavior, such as anxiety, in NW conditions. Anxiety-like behavior was assessed using an elevated plus maze (EPM) by placing mice in the middle between areas and waiting for 5 minutes for mice to explore. The activity was recorded using a video recorder, and the time mice spent in the open arm area was recorded.

### 2.5. Assessment of CRF, POMC, and MC4R mRNA Expression

After behavior test, mice were sacrificed to obtain brain tissue samples. In this research, samples of brain tissue from the amygdala and hypothalamus were dissected using a brain blocker, to obtain the hypothalamus and amygdala areas [27]. The tissue samples obtained were stored in a freezer at −80°C. Subsequently, expression of CRF mRNA in the amygdala, as well as POMC mRNA and MC4R mRNA in the hypothalamus were measured using reverse transcription polymerase chain reaction (PCR). Both amygdala and hypothalamic samples were purified for total RNA using the PureLinkTM RNA Mini Kit (Life Technologies, USA), reverse transcription using the GoScriptTM Reverse Transcription System (Promega, USA), and PCR using GoTaq® DNA Polymerase (Promega). The list of primers used was shown in Table 1.

PCR was carried out using a thermal cycler process, including initial denaturation for 5 minutes at 94°C, followed by 35 cycles of denaturation at 94°C for 40 seconds, annealing at 55°C for 1 minute, extension at 72°C for 2 minutes, and final extension at 72°C for 4 minutes. Subsequently, aliquots from PCR were separated on 2% agarose LE (Promega) using electrophoresis. The gel was stained with ethidium bromide (Sigma–Aldrich) and photographed using ultraviolet transillumination. The band intensity was determined using the ImageJ software.

### 2.6. Data Analysis

This research analyzed percentage changes in food intake and body weight at several time points to assess somatic responses. The data were analyzed using a two-way analysis of variance (ANOVA), followed by Tukey's post-hoc test. Affective response of mice in the open arm area during the anxiety test with the EPM was analyzed using one-way ANOVA, followed by the Tukey's post-hoc test. The relative expressions of CRF mRNA, POMC mRNA, and MC4R mRNA were analyzed using the one-way ANOVA method, followed by Tukey's post-hoc test. Data analysis was performed using the GraphPad Prism 9.0.2 software (GraphPad, Inc., San Diego, CA, USA).

## 3. Results

### 3.1. EGCG Improved NW Conditions-Induced Somatic Behavior Changes in Mice

**Figure 3:**
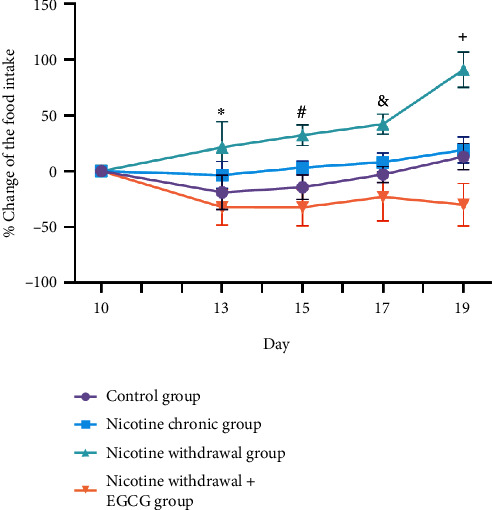
EGCG improved NW conditions-induced somatic behavior changes in mice by ameliorating the percent changes of the food intake. ∗*p* < 0.05 versus NW conditions + EGCG; #*p* < 0.05 versus normal control; *p* < 0.01 NW conditions + EGCG; &*p* < 0.01 versus NW conditions + EGCG; and +*p* < 0.001 versus normal control; *p* < 0.001 versus chronic nicotine; *p* < 0.0001 versus NW conditions + EGCG. Data were shown as mean ± standard error of the mean (SEM; *n* = 6).

**Figure 4:**
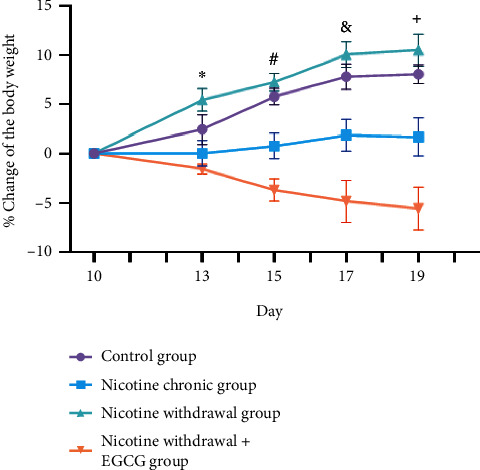
EGCG improved NW conditions-induced somatic behavior changes in mice by ameliorating the percent changes in the body weight. ∗*p* < 0.01 versus NW conditions + EGCG; *p* < 0.05 versus chronic nicotine; #*p* < 0.0001 versus NW conditions + EGCG; *p* < 0.01 versus chronic nicotine; &*p* < 0.0001 versus NW conditions + EGCG; *p* < 0.001 versus chronic nicotine; and +*p* < 0.0001 versus NW conditions + EGCG; *p* < 0.0001 versus chronic nicotine. Data were shown as mean ± SEM (*n* = 6).

The results showed that NW conditions group had a significant increase in food intake and a tendency to gain body weight compared with the normal group. This indicated that NW conditions caused changes in somatic behavior. However, in NW conditions group with EGCG treatment, there was a significant decrease in appetite and weight compared with NW conditions group without EGCG administration. This indicated that EGCG improved NW conditions-induced somatic behavior changes in mice by ameliorating food intake and body weight. The percentage changes in food intake and body weight for each group were shown in Figures 3 and 4.

### 3.2. EGCG Improved NW-Induced Affective Behavior Changes in Mice

**Figure 5:**
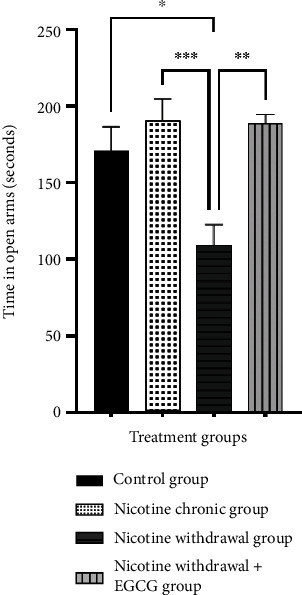
EGCG improved NW conditions-induced affective behavior changes in mice by reducing anxiety-like behavior. EPM data representing anxiety-like behavior were shown as the mean time in open arm ± SEM for 5 minutes (*n* = 6). Statistical analysis was performed using one-way ANOVA followed by Tukey's multiple comparison test. ∗*p* < 0.05; ∗∗*p* < 0.01; and ∗∗∗*p* < 0.001.

Observations on NW conditions-induced affective behavior changes in mice were represented by the time spent by mice in open arms on the EPM instrument. The results showed a significant decrease in the time spent in the open arm in NW conditions group compared with the control. This indicated that NW conditions caused affective changes in anxiety-like behavior. Furthermore, NW conditions group administered EGCG showed a significantly higher time in the open arm than NW conditions group without EGCG treatment. This revealed that the administration of EGCG improved NW conditions-induced affective behavior changes in mice by reducing anxiety-like behavior. The average time in open-arm data for each group was presented in Figure 5.

### 3.3. The Effects of EGCG on CRF, POMC, and MC4R mRNA Expression

**Figure 6:**
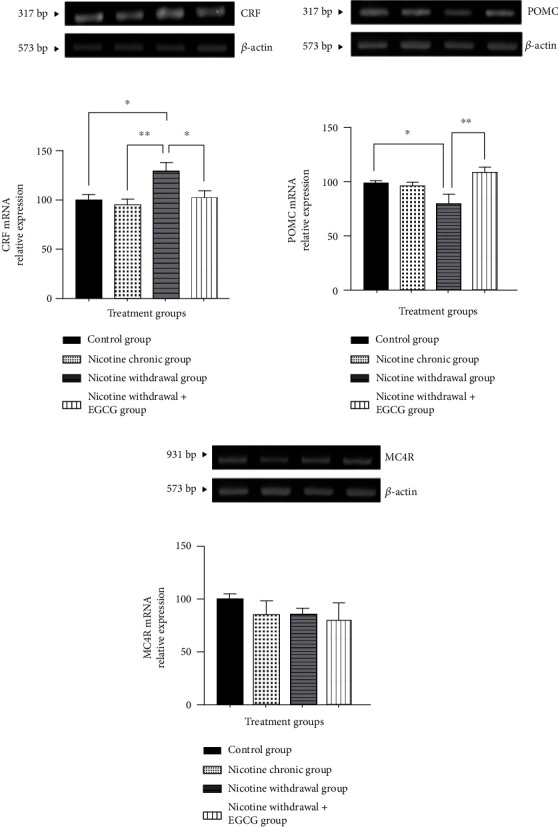
The effects of EGCG on (a) CRF mRNA expression in the amygdala; (b) POMC mRNA expression in the hypothalamus; and (c) MC4R mRNA expression in the hypothalamus of NW conditions mice. ∗*p* < 0.05 and ∗∗*p* < 0.01. Data are shown as mean ± SEM (*n* = 6).

This research found an increase in CRF mRNA expression in the amygdala and a significant decrease in POMC mRNA expression in the hypothalamus in NW conditions group compared with the normal group. The administration of EGCG in NW conditions group inhibited molecular changes in the brain, which decreased CRF mRNA expression in the amygdala and increased POMC mRNA expression in the hypothalamus. Furthermore, there was no significant difference in MC4R mRNA expression in all treatment groups. The average data on CRF, POMC, and MC4R mRNA expression for each group were shown in Figure 6.

## 4. Discussion

The discontinuation of nicotine use after chronic exposure can lead to NW conditions symptoms, such as somatic, affective, and cognitive changes [28]. In this research, NW conditions-induced changes in somatic response, as shown by an increase in body weight and food intake, which indicated the occurrence of overeating. Previous research confirmed that nicotine caused anorexia, hence, after withdrawal, it caused hyperphagia [29]. Furthermore, NW conditions-induced changes in affective response, as indicated by anxiety-like behavior [30].

EGCG at a dose of 50 mg/kg in mice was a proven polyphenolic compound found abundantly in green tea, which effectively reduced the reward effect caused by nicotine [26]. The compound exhibited pharmacological effects as anti-obesity and also produced neuroprotective and neurodegenerative effects against depression as well as stress-induced behavior disorders [21, 22]. However, the effects of EGCG on NW had not been researched. Therefore, this research aimed to investigate whether EGCG at a dose of 50 mg/kg can inhibit NW symptoms.

The results showed a significant increase in body weight during NW compared with normal conditions, which continued until the last day of observation. Similarly, a previous report found that weight gain during NW conditions can persists for up to 14 days [31]. According to another research, nicotine was a major component of cigarette smoke, which reduced appetite [32]. However, this research discovered that chronic nicotine conditions did not reduce appetite. The variation in results can be attributed to the different methods used in various research.

In addition to affective changes associated with NW, such as anxiety, sudden cessation of chronic nicotine administration in NW conditions group caused anxiety-like behavior, as indicated by a significant decrease in the time spent by mice in the open arms. A previous report suggested that NW conditions can cause behavior changes resembling depression-like states [33]. Moreover, anxiety was often accompanied by several painful symptoms, which can trigger or exacerbate anxiety and depression [34]. Another research found that long-term nicotine exposure and withdrawal resulted in hyperalgesia with a reduction of GAD67, leading to a GABA decrease [35].

This research found that administering EGCG in mice at doses of 50 mg/kg at 07.00 am and 05.00 pm inhibited the increase in food intake and body weight during NW conditions. This was in line with previous research, where EGCG 50 and 100 mg/kg per day given to mice together with a high-fat diet (HFD) for 20 weeks reduced body weight and adipose tissue [36]. However, in another research, the administration of EGCG at a lower dose of 12 mg/kg (07.00 am) and 24 mg/kg (07.00 am) reduced overeating in rats induced by a HFD [37].

The measurement of anxiety-like behavior showed that administering EGCG 50 mg/kg reduced anxiety-like behavior in mice experiencing NW conditions. This was represented by a significant increase in time spent in the open arm in NW conditions group that was given EGCG post-treatment. Similarly, a previous report stated EGCG, as an anxiolytic at a dose of 7.5–60 mg/kg increased the time spent by mice in the open arm [38]. This research found that EGCG inhibited behavior changes in NW conditions.

In addition to changes in behavior during NW, molecular changes were also observed in the hypothalamus and amygdala of the brain. Molecular mechanism in the amygdala region represented anxiety and stress. Moreover, stress alter the sensitivity of glucocorticoid receptors in the nervous system, cause HPA axis and autonomic dysfunction, leading to high production and release of pro-inflammatory cytokines. The pathophysiological pathway involved changes in neurotrophic support and neuron–glia interaction, resulting in central sensitization to pain, which was linked to the same pathway in depressive and anxiety symptoms [34, 39]. Glucocorticosteroids in the brain played an important physiological process involved in various stress-related conditions to preserve neuroplasticity within the hippocampus [40]. Research had established that the neurohormonal stress axis involving sympathoadrenal, and HPA activation played a crucial role in the onset and progression of alcoholic neuropathy [41].

The lack of activity of the dopaminergic system and hyperactivity of CRF in the HPA caused anxiety-like behavior [4]. CRF played a central role in developing negative affective states, such as anxiety-like behavior, after nicotine discontinuation [7, 42]. In this research, the CRF mRNA expression in the amygdala increased under NW conditions. This was consistent with previous research, where CRF mRNA was overexpressed in CeA during NW conditions [43, 44]. CRF was secreted from the hypothalamus and stimulated adrenocorticotropic hormone, which induced the release of corticosterone and glucocorticoids from the adrenal cortex [33, 45, 46]. The upregulation of glucocorticoids suppressed the expression of brain-derived neurotrophic factor (BDNF), leading to BDNF hypofunction [47]. EGCG also inhibited stress-stimulated HPA axis dysfunction and decreased the expression of CREB and BDNF [21]. Similarly, in this research, EGCG decreased CRF mRNA expression in the amygdala under NW conditions by increasing BDNF and thereby, inhibiting HPA axis dysfunction.

The ARC of the hypothalamus contained an anorexigenic neuropeptide, namely POMC, which played a role in suppressing appetite [48, 49]. Additionally, the PVN of the hypothalamus contained MC4R, an endogenous peptide with the type G protein-coupled receptor as the main agent controlling appetite [50]. This research showed that under NW conditions, there was a decrease in POMC mRNA expression in the hypothalamus compared with normal conditions but not with MC4R mRNA expression. Similarly, a previous investigation stated that exposure of mice to 0.6 mg/kg/day of nicotine for 6 days did not significantly increase MC4R expression in the lateral hypothalamic area, but was effective in the prefrontal cortex [51].

The administration of EGCG at a dose of 50 mg/kg in mice twice a day during NW conditions increased POMC mRNA expression in the hypothalamus of mice. A previous research on the effect of EGCG on obese mice models also showed that the anti-obesity activity of EGCG was produced through increased POMC expression in the hypothalamus of mice [24]. Therefore, the reduction in the food intake and body weight in this research was due to mechanism of EGCG in increasing POMC mRNA expression in the hypothalamus of mice under NW conditions. Another report found that EGCG treatments under normal conditions also increased POMC mRNA expression in the hypothalamus and strongly suppressed body weight gain and fat accumulation in mice [24].

The absence of changes in MC4R mRNA expression in the hypothalamus was due to the regulation of energy expenditure during NW conditions by the MC4R gene in the paraventricular hypothalamus. Therefore, it can be assumed that the regulation of energy expenditure in energy balance was not only mediated by the MC4R gene in the paraventricular hypothalamus but also mediated by other pathways. Other research also showed that appetite control was influenced by signaling from the periphery by several hormones, such as leptin, ghrelin, and insulin [50].

This research was the first to explore the effectiveness of EGCG in overcoming NW conditions and its underlying mechanism, particularly in the amygdala and hypothalamic melanocortin system pathway repair. The potential of EGCG to reduce effective and somatic behavior changes in NW conditions was also examined. Although sufficient results were obtained from NW and NW + EGCG groups, there were still limitations, challenges, and opportunities for further research. For example, the absence of a normal EGCG group in this research limited its comprehensiveness. Therefore, further investigations analyzing the normal EGCG group in NW research were recommended to strengthen the results obtained.

Furthermore, research related to optimization must also be conducted by using a variety of doses to obtain the minimum dose needed to reduce affective and somatic behavior changes in NW conditions. Investigating the direct effect of EGCG on the plasma nicotine level in the blood can also reveal another potential use of EGCG. Several challenges and opportunities still needed to be investigated to explore the underlying mechanism of EGCG in other peptide systems in the hypothalamus, such as orexin, melanin-concentrating hormone, cocaine- and amphetamine-regulated transcript, and neuropeptide Y. This research was the first to successfully confirmed that the administration of EGCG at a dose of 50 mg/kg in mice twice a day inhibited NW. Therefore, these results formed the basis for further scientific developments to explore the effect of EGCG on molecular responses involving the melanocortin system in the hypothalamus and CRF in the amygdala for other diseases.

## 5. Conclusions

This research showed that EGCG inhibited affective, somatic, and molecular changes in NW conditions. Affective changes were related to emotions, such as anxiety and depression. Somatic changes included physical changes, namely increased appetite, which contributed to body weight gain during chronic NW conditions. In affective changes, EGCG reduced anxiety-like behavior in mice experiencing NW conditions. Meanwhile, in somatic changes, EGCG inhibited the increase in food intake and body weight during NW conditions. Molecular mechanism of EGCG in inhibiting affective and somatic changes in NW conditions was mediated by decreasing CRF mRNA expression in the amygdala and increasing POMC mRNA expression in the hypothalamus.

## Figures and Tables

**Table 1:** Temperature and wildlife count in the three areas covered by the research.

Genes	Primer sequence	PCR product (bp)
*CRF F*	5′-GAAGAGAAAGGGGAAAGGCAAAGA-3′	202
*CRF R*	5′-GCGGTGAGGGGCGTGGAGTT-3′	
*POMC F*	5′-GCTTGCATCCGGGCTTGCAAACT-3′	317
*POMC R*	5′-AGCAACGTTGGGGTACACCTT-3′	
*MC4R F*	5′-TTAATACCTGCTAGACAACTCA-3′	931
*MC4R R*	5′-ATGTATACTTCCCTCCACCTCTG-3′	
*β-Actin F*	5′-TGTTACCAACTGGGACGACA-3′	573
*β-Actin R*	5′-AAGGAAGGCTGGAAAAGAGC-3′	

## Data Availability

The data that support the findings of this study are available on request from the corresponding author (Mahardian Rahmadi).
